# Molecular Identification and Characterization of *Vibrio* Species and *Mycobacterium* Species in Wild and Cultured Marine Fish from the Eastern Mediterranean Sea

**DOI:** 10.3390/microorganisms8060863

**Published:** 2020-06-07

**Authors:** Yael Regev, Nadav Davidovich, Ran Berzak, Stanley C. K. Lau, Aviad P. Scheinin, Dan Tchernov, Danny Morick

**Affiliations:** 1 Department of Marine Biology, Leon H. Charney School of Marine Sciences, University of Haifa, Haifa 3498838, Israel; yaelchac@gmail.com (Y.R.); ranberzak@gmail.com (R.B.); shani.aviad@gmail.com (A.P.S.); dtchernov@univ.haifa.ac.il (D.T.); 2 Morris Kahn Marine Research Station, University of Haifa, Haifa 3498838, Israel; 3 Israeli Veterinary Services, Bet Dagan 5025001, Israel; Nadavd@moag.gov.il; 4 Department of Ocean Science, The Hong Kong University of Science and Technology, Clear Water Bay, Kowloon, Hong Kong; scklau@ust.hk

**Keywords:** *Vibrio*, *Mycobacterium*, zoonotic microorganisms, Mediterranean Sea, wild fish, farmed fish

## Abstract

In contrast to numerous documented pathogens and infectious diseases of aquaculture, there is a lack of baseline data and information regarding pathogenic agents’ prevalence in wild marine fish populations. This study focused on two common fish pathogenic microorganisms, namely *Mycobacterium* species and *Vibrio* species, both of which are known to be major causes of fish loss, occasionally to the extent of being a limiting factor in fish production. Both microorganisms are known as zoonotic agents. In total, 210 wild marine indigenous and Lessepsian fish from four different species from the eastern Mediterranean Sea were sampled and tested for *Vibrio* species and *Mycobacterium* species during a two-year period (2016–2017). Using PCR with 16S rRNA primers, we detected different strain variations of *Mycobacterium* species and *Vibrio* species and, based on the sequencing results, the overall prevalence for *Vibrio* species in wild fish in 2016 was significantly higher compared to 2017. No significant difference was detected for *Mycobacterium* species prevalence in wild fish between 2016 and 2017. In addition, 72 gilthead seabream (*Sparus aurata*) from an Israeli offshore marine farm were also examined during the two-year period (2017–2018). The results suggest that *Mycobacterium* species prevalence was significantly higher in 2018, while in 2017 there was no positive results for *Mycobacterium* species. In addition, there was no significant difference between both years in regard to the prevalence of *Vibrio* species for maricultured fish. These results highlight the necessity of continuous molecular monitoring in order to evaluate the prevalence of pathogenic microorganisms in both wild and cultured fish populations.

## 1. Introduction

Over the past few decades, there has been a worldwide increase in reports of diseases affecting marine organisms of different taxa [1]. Climate change is affecting the marine ecosystem, which is already subjected to many anthropogenic disturbances, such as overfishing, pollution and habitat destruction. Climate warming can increase pathogen development and their survival rates, disease transmission and host susceptibility [2]. Environmental conditions play a crucial role not only in pathogen transmission, but also as risk factors for the occurrence of clinical diseases. Unlike mammals that regulate their internal environments, most fish are poikilotherms with little ability to regulate their core body temperature. In this situation, both the microbe and the host are physiologically tied to the environment they live in and have an optimal temperature range for survival. Extended periods outside the optimal range usually results in death [3]. This is especially true in the Mediterranean Sea, which is one of the biggest reservoirs of biodiversity in the world [4]. Diverse cold and tropical marine fauna combine and mix due to the basin’s oceanographic and biogeographical properties and, therefore, might serve as “miniature model” for the world’s oceans and provide insights into global patterns of marine ecosystems [5].

Although aquaculture production has increased dramatically, fish consumption still largely depends on fisheries [6]. Wild fish have an important ecological role in the ecosystem and economic role as a major protein source for humans [7]. A wide range of marine pathogens from aquaculture is well documented, but there remains a lack of baseline data and information regarding pathogenic agents’ prevalence in the wild fish population [8]. On the other hand, aquaculture is a fast-growing industry for the production of high protein-sourced foods, including fish production [9]. This growth is accompanied by concerns from both the public and private sectors [9], as fish production is commonly associated with serious environmental impacts (e.g., water pollution, pathogen transmission, and temperature changes). Mariculture production is usually as crowded and intensive production ponds. Fish are routinely treated and harvested from the walkway or raft around the cages, and boats are used for transportation of feed and for harvest [10]. In Israel, there are several offshore marine farms along the Israeli Mediterranean coastline. Gilthead seabream (*Sparus aurata*) is the dominant mariculture species, with an annual production in 2018 of 2450 tons, and 2310 tons in 2019 [11]. Global aquaculture production has constantly increased during the last five decades [12]. Although aquaculture in the Mediterranean is considered a relatively young industry, finfish diseases have been reported to cause substantial problems and mortalities among farmed stocks [13]. Two apparent reasons that play a central role in the transmission of infectious pathogens are the farming activity, and the open design of Mediterranean aquaculture systems. As such, the transport of infected farmed fish from hatcheries, infected equipment, staff, and vessels, as well as through water currents, has been the main focus of fish health and biosecurity programs [13]. Infectious agents are also common in marine waters. Bacteria and viruses can be transmitted both horizontally and vertically, due to oceanographic conditions in nearshore environments that are strongly influenced by local conditions [14], and they can reduce commercial species’ growth and survivorship or decrease seafood quality. These impacts seem most problematic in the stressful and crowded conditions of aquaculture, which increasingly dominates seafood production as wild fishery production plateaus [15]. It is often difficult to accurately estimate the impacts of diseases on wild populations, especially those of pelagic and subtidal fish species. However, there are a few quantitative data demonstrating that wild species near farms suffer more from infectious diseases than those in other areas. The movement of exotic infectious agents to new areas continues to be a great concern. It is important to note that the exposure of a host to a pathogen does not always result in infection, which, in turn, does not always lead to disease [16]. Moreover, infection may occur geographically far from the immediate vicinity where infection is either detected or disease first becomes evident. These variables make diagnosis and/or pathogen evasion highly problematic.

Zoonotic microorganisms present a global public health concern. Those pathogens are known to cause protracted illness, especially in immuno-compromised individuals [17]. As aquaculture production and the consumption of aquaculture products increase, the possibility of contracting zoonotic infections from either handling or ingesting these products also increases. Disease outbreaks are often related to management factors, such as the quality and quantity of nutrients in the water and high stocking density, which can increase bacterial loading on the external surface of the fish. As a result, diseased fish are more likely to transmit infection to humans [18].

This research focused on two types of bacterial pathogens, namely *Vibrio* and *Mycobacterium*, which are known as zoonotic pathogens [17] and are major causes of fish mortality [19]. *Vibrio* species are Gram-negative curved rods that occur naturally in marine, estuarine, and freshwater systems worldwide. They occupy habitats ranging from the deep sea to shallow aquatic environments [20]. More than 70 different *Vibrio* species are known, 12 of which are recognized as human pathogens [21]. Some species include human and animal pathogens capable of causing gastroenteritis, wound infections, cholera, and fatal septicemia [22]. Infections caused by *Vibrio* have been observed and documented in marine and estuarine-type fishes. The disease is known to have increased death rates (>50%) in fish farms soon after an outbreak [23]. Fish vibriosis usually start with sluggishness and a loss of appetite. The disease may cause discoloration and eventual decay of the skin. Swollen sores may appear on the body, and in many cases protrude through the skin surface. Another symptom is redness around the fins and mouth. When the disease becomes systemic, it can cause exophthalmia, and the gut and rectum may be bloody and filled with fluid [23]. *Vibrio* sp. cause some of the most significant infections of marine finfish. All marine fish are probably susceptible to at least one species. *Vibrio* sp. have been infrequently isolated from freshwater aquarium fish and freshwater salmonids that have been fed marine offal [24]. *Vibrio* sp. are typically facultative pathogens that can readily survive and multiply in the environment, although the relative pathogenicity of environmental versus fish isolates is uncertain. *Vibrio* sp. are usually isolated from the mucosal surfaces and internal organs of clinically healthy fish. The highest environmental prevalence is in organically-polluted water and high salinity. A major predisposing risk factor for most types of fish-vibriosis is high water temperature. Crowding, organic pollution, and other stressors can also precipitate diseases outbreaks. *Vibrio* strains also vary considerably in virulence, and some of them can cause disease without any predisposing stress. Some *Vibrio* species produce hemolysins (which may cause anemia) and proteases (which may cause muscle damage) [24].

Mycobacteria (Family *Mycobacteriaceae*) are pleomorphic, Gram-positive, acid-fast, aerobic, non-motile rods [17]. Fish mycobacteriosis is caused by non-tuberculous mycobacteria (NTM), and considered among the most chronic diseases occurring in aquatic animals [25]. NTM is a group of more than 150 different species with distinct virulence features [26]. In addition, fish mycobacteriosis is a chronic disease that has substantial economic consequences, as infections may significantly decrease production and trade. Some fish NTM are highly virulent and zoonotic [17]. Typical signs of *Mycobacterium* in fish are weight loss or emaciation, scale loss, ulcerations or hemorrhage along the body wall, granulomas, poor appetite and attitude, and often a history of reproductive problems [27]. Both external and internal clinical signs caused by each pathogen are dependent on the host species, age of the fish, and stage of the disease (acute, chronic, and sub-clinic carrier); the signs are not always correlated or present at all [28]. *Mycobacterium marinum* was first isolated in 1926 from several marine fish species [24]; it is the most common etiological agent of fish mycobacteriosis [24]. Other significant mycobacteria reported in marine fish are *Mycobacterium chelonae* and *Mycobacterium fortuitum*. NTM are saprophytes that reside in both soil and water, where they can survive for years. Commonly, gross examination of infected fish reveals grayish to white granulomatous nodules in various internal organs, mainly the spleen, liver, and kidney. However, in some cases, external signs may be present in the form of dermal ulceration [24]. The objectives of this study were: (a) to evaluate the prevalence of *Vibrio* sp. and *Mycobacterium* sp. in wild and cultured marine fish species; (b) to compare pathogen prevalence between different years in farmed and wild fish; (c) to compare pathogen prevalence in different internal organs and maturity age; (d) to identify different strain variations of *Vibrio* sp. and *Mycobacterium* sp. in wild and cultured fish; and (e) to assess transmission of the pathogens from wild to cultured fish and vice versa.

## 2. Materials and Methods

### 2.1. Fish and Tissue Sampling

**Figure 1:**
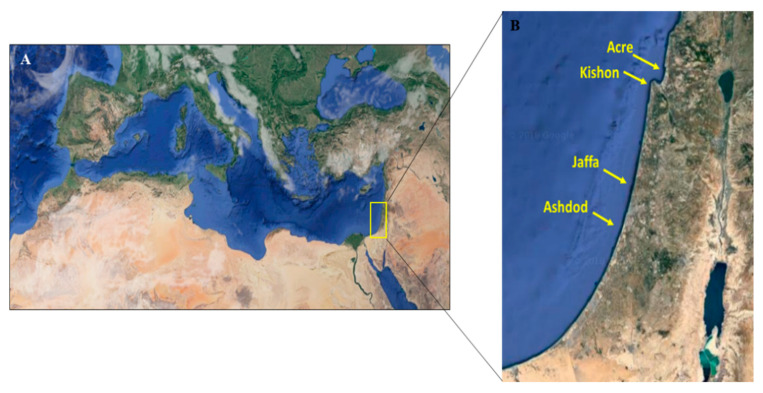
Geographical distribution of the sampling locations described in this study: (**A**) sampling area is in the most eastern basin of the Mediterranean Sea; and (**B**) fish were sampled from four fishing ports along the Israeli coast—two in the north (Acre and Kishon) and two in the south (Jaffa and Ashdod). Template map source: Google Earth.

Four wild fish species: striped red mullet (*Mullus surmuletus*), Randall’s threadfin bream (*Nemipterus randalli*), lizardfish (*Saurida lessepsianus*), and round sardinella (*Sardinella aurita)* were caught by trawlers and fishermen. Fish were collected at four ports along the Israeli Mediterranean shoreline: Acre, Kishon, Jaffa, and Ashdod (Figure 1). In addition, gilthead seabream (*Sparus aurata*) specimens were sampled during their growth period from a fish farm located 12 km offshore in the southern area of the Israeli Mediterranean Sea. All wild specimens were sampled during 2016–2017, and the cultured *S*. *aurata* were sampled during 2017–2018 (Table 1 and Table 2). Fish were placed on ice immediately on the boat and transferred to the laboratory where weight, total length, and visual inspections were carried out. It should be mentioned that the fish were obtained at the ports and nearby fish markets, but the exact fish capture sites are not recorded in this study. All specimens were aseptically dissected for tissue sample collection according to fish necropsy protocol [29]. All samples were kept frozen at −80 °C until further analysis. From each fish specimen, liver and kidney tissues were collected and kept frozen at −80 °C until use.

### 2.2. Comparison of Kidney and Liver Tissues

In total, 251 specimens were taken from the kidneys and livers of the five fish species (four wild marine fish species and cultured *S*. *aurata*). These were analyzed and compared to determine *Vibrio* sp. and *Mycobacterium* sp. specific tissue prevalence. Only specimens tested for both tissues were included in the statistical analysis.

### 2.3. Comparison of Juvenile and Adult Fish

In total, 150 mature fish and 95 juveniles, from four fish species (only mature *M. surmuletus* were tested, and therefore were not tested for statistical significance), were analyzed and compared for *Vibrio* sp. and *Mycobacterium* sp. prevalence. The separation of mature and juvenile fish was determined by the total length: *N. randalli* reach sexual maturity at a total length of ~110 mm [30]; *S. aurita* at a total length of ~155 mm [31]; *S. lessepsianus* at a total length of ~180 mm [32]; and *S. aurata* at a total length of ~260 mm [33].

### 2.4. DNA Extraction

Extraction of total DNA from the tissues of each specimens was performed using the Wizard SV Genomic DNA Purification System kit (Promega Corporation, Madison, WI, USA) and in according to the manufacturer’s specifications. Up to 20 mg of tissue sample were placed in 275 µL of manufacturer’s Digestion Solution Master Mix, followed by an overnight incubation in 55 °C (16–18 h), then a 250 µL of Lysis buffer was added. For purification of DNA, the lysates were transferred to the manufacturer’s mini-column assembly, centrifuged for 3 min at 13,000× *g*, followed by four subsequent centrifuges at 13,000× *g* for 1 min with 650 µL wash solution. DNA was eluted using a 2-min incubation with 250 µL nuclease-free water and 13,000× *g* centrifuge for 2 min. The DNA concentration and quality were determined at 280 nm with a NanoDrop One (NanoDrop Ins., Thermo Scientific), and the extracted DNA were stored at −20 °C until further analysis.

### 2.5. PCR Amplification

PCR for both *Vibrio* sp. and *Mycobacterium* sp. were performed in reaction tubes preloaded with 3 µL of DNA, 0.2 µL of each primer, 9.1 µL of ultra-pure PCR water, and 12.5 µL GoTaq Green Master mix (Promega Corporation, Madison, WI, USA) on a SimpliAmp Thermal Cycler (Applied Biosystems, Foster City, CA, USA). The amplification reaction for *Mycobacterium* 16S rRNA was subjected to 40 cycles (4 min at 95 °C, 30 s at 62 °C, and 30 s at 72 °C), followed by 10 min of extension at 72 °C. Primers T39 5′-GCGAACGGGTGAGTAACACG-3′ and T13 5′-TGCACACAGGCCACAAGGGA-3′ amplified a 924-bp segment [34]. The amplification reaction for *Vibrio* 16S rRNA was subjected to 40 cycles (4 min at 95 °C, 30 s at 61 °C, and 30 s at 72 °C), followed by 10 min of extension at 72 °C. Primers 63f 5′-CAGGCCTAACACATGCAAGTC-3′ and 763r 5′-GCATCTGAGTGTCAGTATCTGTCC-3′ amplified a 700-bp segment [35].

### 2.6. Sequencing and Phylogenetic Analysis

*Vibrio* PCR (primer “63f”) and *Mycobacterium* PCR (primer “T39”) amplicons were purified by ExoSAP-IT (Affymetrix, Santa Clara, CA, USA) and sequenced by Sanger sequencing method (Hy-Labs, Rehovot, Israel). All sequences were aligned and compared to representative sequences available in Arb-Silva website and in GenBank by BLAST using the BioEdit Sequence Alignment Editor and MEGA10 software. Phylogenetic trees were visualized with the MEGA10 software. Robustness of nodes on the phylogeny was assessed by 1000 bootstrap replicates using Maximum Parsimony analysis. All sequences from positive samples were deposited in GenBank and accession numbers are provided in Tables S1 and S2.

### 2.7. Statistical Analyses

All statistical analyses were conducted using IBM SPSS Statistics for Windows, version 20.0 (IBM Corp., Armonk, NY, USA). Multiple logistic regression analysis was chosen to analyze the various effects on *Vibrio* sp. and *Mycobacterium* sp. prevalence. For all tests, a *p*-value of <0.05 was considered significant.

## 3. Results

### 3.1. Vibrio sp.

**Figure 2:**
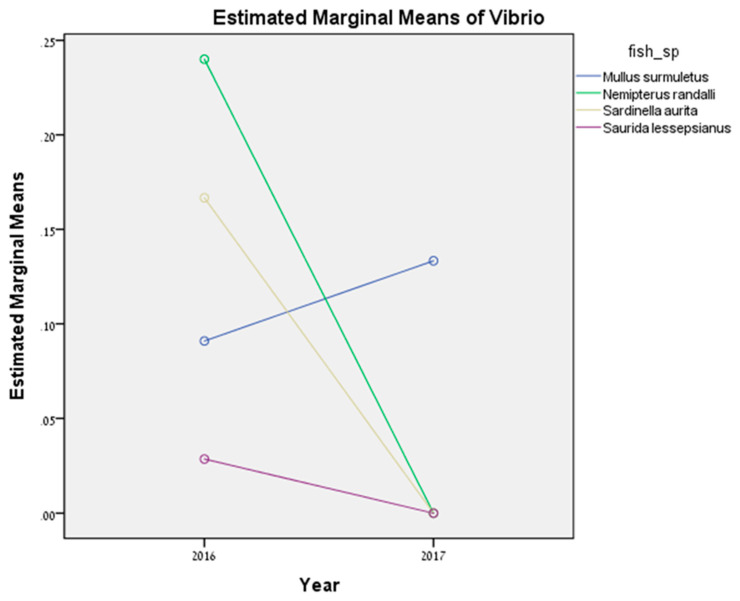
*Vibrio* sp. prevalence interaction between fish species and between years.

In total, 113 wild fish in 2016 and 97 in 2017 were tested for *Vibrio* sp. by PCR amplification (Table 3). In addition, 45 cultured fish (*S. aurata*) in 2017 and 27 in 2018 were also tested (Table 4). All visually inspected fish showed no external or internal alterations. Based on the sequencing results in 2016, the total prevalence of positive results for *Vibrio* sp. in wild fish was significantly higher compared to 2017 (F = 5.91, *p* = 0.031). Figure 2 shows the significant effect of interaction between fish species and years (F = 2.68, *p* = 0.048). *N. randalli*, *S. aurita,* and *S. lessepsianus* exhibited a decrease in *Vibrio* sp. prevalence in 2017. However, *M. surmuletus* showed an increase in *Vibrio* sp. prevalence in 2017. As for the cultured fish farm, no change in prevalence was found between the years (*p* > 0.05). In 2017, the total prevalence of positive results for *Vibrio* sp. was 8.89%, compared to 2018 when the prevalence for *Vibrio* sp. was 3.7%. In total, 251 samples were collected from kidney and livers from all the five species and were tested for *Vibrio* sp. (Table 5 and Table 6). The kidney and liver samples do not differ significantly (*p* > 0.05). From kidney samples, positive results were obtained in two samples of *M. surmuletus* (5.4%), six *N. randalli* (9.7%), two *S. aurita* (4.5%), one *S. lessepsianus* (2.1%), and two *S. aurata* (5.2%) (Table 3). From liver samples, positive results were obtained in two samples of *M. surmuletus* (5.4%), two *S. aurita* (4.5%), and three *S. aurata* (4.9%) (Table 3). No positive results were obtained in *N. randalli* and *S. lessepsianus* liver tissues. Two specimens of *M. surmuletus* exhibited presence of *Vibrio* sp. in both the liver and kidney tissues. In total, 150 mature fish and 95 juveniles from four fish species (only mature *M. surmuletus* were tested, and therefore this species was not included in the statistical analysis) were analyzed and compared for *Vibrio* sp. prevalence (Table 5, Table 6). No positive results were obtained in *N. randalli* and *S. aurita* juveniles, while positive mature fish of the same species showed a prevalence of 12.5% and 6.1%, respectively. A positive result was obtained for one juvenile *S. lessepsianus* (25%) and higher prevalence was found in juvenile *S. aurata* (7.7% of juvenile compared to 5% of mature specimens).

**Figure 3:**
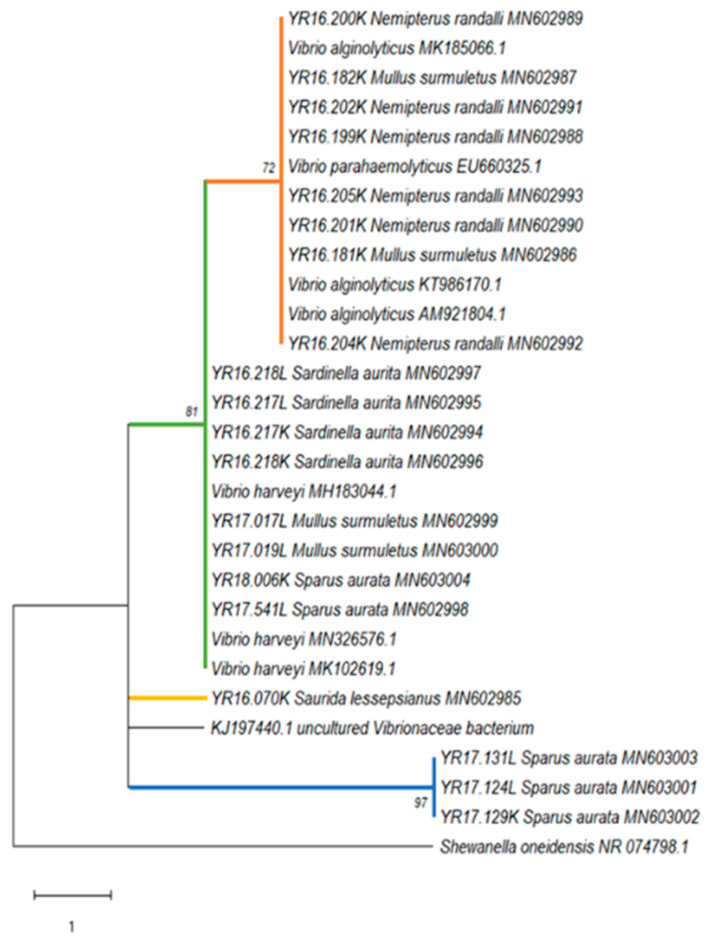
*Vibrio* sp. phylogenetic tree. Maximum Parsimony analysis phylogenetic tree of the *Vibrio* sp. derived from 16S rRNA gene partial sequences. The sequence name for positive samples of this study begins with YR and includes the capture year, identification number, tissue (L, liver; K, kidney), host species, and GenBank accession number. The tree was rooted by using *S. oneidensis* as the outgroup. Numbers on the branches indicate bootstrap proportions (1000 replicates, only values ≥ 70% are reported). Available GenBank and Arb-Silva accession numbers are shown. The scale bar represents one nucleotide substitution per site.

The phylogenetic tree constructed from the 16S rRNA gene partial sequences (Figure 3) revealed a similarity within four different groups of *Vibrio* sp.: six *N. randalli* and two *M. surmuletus* segments from wild Mediterranean Sea fish shared an identical nucleotide sequence and displayed similarity to *V. alginolyticus* and *V. parahaemolyticus* (orange group, Figure 3). All these segments were detected from kidney tissues in 2016. The second group (green group, Figure 3) showed similarity to *V. harveyi* and contained two samples of *S. aurita* from 2016, both segments of which were from kidney and liver tissues, two 2017 *M. surmuletus* (liver tissue), and two cultured Mediterranean Sea fish *S. aurata* (one from 2017 and one from 2018). The third group contained one strain from 2016 *S. lessepsianus* which did not show any similarity to specific *Vibrio* species (yellow group, Figure 3). Three *S. aurata* samples from 2017 from cultured Mediterranean Sea fish also did not showed any similarity to specific *Vibrio* species (blue group, Figure 3).

### 3.2. Mycobacterium sp.

**Figure 4:**
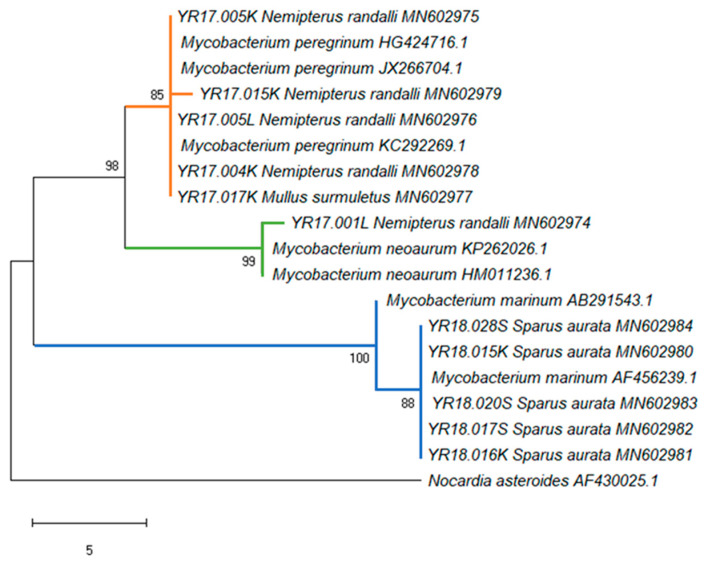
*Mycobacterium* sp. phylogenetic tree. Phylogenetic tree output from the Maximum Parsimony analysis of the *Mycobacterium* sp. derived from 16S rRNA gene partial sequences. The sequence name for positive samples of this study begins with YR and includes the capture year, identification number, tissue (L, liver; K, kidney; S, spleen), host species, and GenBank accession number. The tree was rooted by using *N. asteroides* as outgroup. Numbers on the branches indicate bootstrap proportions (1000 replicates, only values ≥70% are reported). Available GenBank and Arb-Silva accession numbers are shown. The scale bar represents five nucleotide substitution per site.

In total, 113 wild fish in 2016 and 97 in 2017 were tested for *Mycobacterium* sp. by PCR amplification (Table 7). In addition, 45 cultured fish (*S. aurata*) in 2017 and 27 in 2018 were tested (Table 8). Based on the sequencing results, in the 2016 study, no positive results were recorded in wild fish, compared to 2017 when the overall prevalence for *Mycobacterium* sp. was 5.15%. The only species that showed the presence of this pathogen were *M. surmuletus* (6.67%) and *N. randalli* (9.52%) (Table 7). According to the multiple logistic regression, no significant difference in prevalence was found over the total prevalence of *Mycobacterium* sp. between the years. As for the farmed fish, a significantly higher prevalence (F = 9.943, *p* = 0.002) was found in 2018 (18.52%) compared to 2017, in which there were no positive results for *Mycobacterium* sp. (Table 8). In total, 251 samples were taken from the kidney and livers of each specimen from all the five fish species and tested for *Mycobacterium* sp. (Table 9). A significantly higher prevalence was found in kidney samples compared to liver tissues (F = 2.518, *p* = 0.041). From the kidney samples, positive results were obtained in one specimen of *M. surmuletus* (2.7%), four *N. randalli* (6.5%), and two *S. aurata* (3.3%). From liver samples, positive results were only obtained in two *N. randalli* (3.2%) (Table 10). No positive results were obtained in *S. aurita* (Table 2) and *S. lessepsianus* tissues. One specimen of *N. randalli* exhibited presence of *Mycobacterium* sp. in both the liver and kidney tissues. In total, 150 mature fish and 95 juveniles from four fish species (only mature *M. surmuletus* were tested and thus were not included in the statistical analyses) were analyzed and compared to ascertain *Mycobacterium* sp. prevalence (Table 10). No positive results were obtained in both mature and juvenile *S. aurita* and *S. lessepsianus*. Higher prevalence was observed in mature *N. randalli* and *S. aurata* compared to their juvenile counterparts. There was no significant difference (*p* > 0.05) in total prevalence of *Mycobacterium* sp. between the mature and the juvenile. The phylogenetic analysis of *Mycobacterium* segments revealed similarity across three different groups of *Mycobacterium* (Figure 4): three 2017 *N. randalli* and one *M. surmuletus* segments from wild Mediterranean Sea fish shared an identical nucleotide sequence (YR17.015K differed by one nucleotide) and showed similarity to *M. peregrinum* (orange group, Figure 4). *N. randalli* YR17.001L (green group, Figure 4) showed similarity to *M. neoaurum*, only differing from the orange group by nine nucleotides. All five segments from the 2018 fish farmed *S. aurata* were identical to each other, were similar to *M. marinum* (blue group, Figure 4), and differed from the orange group by 21 nucleotides.

## 4. Discussion

*Vibrio* sp. and *Mycobacterium* sp. are widespread in nature, especially in the marine environment. Fish infected by these bacteria could be a source of zoonotic risk for human health [26], and are known to cause infections in humans with different degrees of severity, especially in immunocompromised individuals [22,36]. Although *Vibrio* sp. and *Mycobacterium* sp. have been detected and studied in many fish species around the world, including various wild marine fish species [21,37,38,39,40,41], at the time of writing, this is the first study to identify *Vibrio* sp. in the Lessepsian migrant species *Saurida lessepsianus* and the endemic Mediterranean fish *Sardinella aurita.*

In this study, the presence of two important aquatic bacteria were investigated in four marine wild fish and one cultured farm fish in the eastern Mediterranean Sea. Both pathogens were detected in indigenous and Lessepsian species, and their prevalence varied greatly between fish species. In both pathogens, there were no statistically significant differences between the different wild species. However, the overall prevalence of *Vibrio* sp. was significantly higher in 2016 compared to 2017. *Vibrio* species are omnipresent and widely distributed in aquatic environments all over the world [42]. The occurrence of *Vibrio* sp. In fish, is commonly associated with elevated temperature, especially in temperate climes. Generally, *Vibrio* species are detected in summer but are less common in winter, whereas the variations of *Vibrio* sp. population size are lower in tropical and subtropical waters [43]. Reports have shown a significant association between rising seawater temperature and an increase in the number of *Vibrio* sp. infections, suggesting that global warming could be a factor in the emergence of vibriosis in temperate areas, due to its influence on resident bacterial communities [43]. In 2016, during a continuous survey, most of the positive samples were detected in the summer period, which can explain the higher prevalence of *Vibrio* sp. in 2016. However, most of the surveys in 2017 were between October and December, and the prevalence was lower. Within the cultured fish species, in 2018, the prevalence for *Mycobacterium* sp. in *S. aurata* was significantly higher compared to 2017, where no infection was detected at all. Due to logistical difficulties in field sampling (a seasonal ban on fishing vessels), we could not sample each season more than once for appropriate comparison. Thus, we cannot definitely determine if the prevalence of *Vibrio* sp. is related to temporal changes in exposure or due to temperature shifts. Hence, this is a limitation of this study. The total prevalence for both pathogens was higher in the Lessepsian fish *N*. *randalli* and in the indigenous *M. surmuletus,* with no reported clinical signs. High prevalence in an asymptomatic fish may indicate they can serve as carriers and horizontally infect other susceptible species living in proximity [44]. *N*. *randalli* is an invasive species, first reported in the Mediterranean in 2005 [45], and has become a dominant fish species in the Israeli ichthyofauna within the past five years [46]; the reasons for its successful establishment are unknown [47]. Although *Vibrio* sp. and *Mycobacterium* sp. can be isolated from different organs (e.g., spleen and liver), it has been suggested that these pathogens have an affinity for, or are better detected, in kidney tissue [48,49]. This is in agreement with our results, where the prevalence for both pathogens in kidney was higher than in liver tissues in most of the examined fish species. In addition, the comparison of *Vibrio* sp. and *Mycobacterium* sp. prevalence in juvenile and adult fish was examined in four out of the five fish species (only mature *M. surmuletus* were detected in this study and therefore were not included in the statistical analysis). In the wild fish, the highest prevalence was detected in adult-stage specimens. However, as for the cultured fish *S. aurata,* positive results were obtained in both adult and juvenile specimens. It seems that at least the fish species tested in this study in different life stages are similarly susceptible for infection of *Vibrio* sp. and *Mycobacterium* sp. [50].

Phylogenetic analyses, based on the 16S rRNA gene sequences, revealed that all detected *Vibrio* strains were divided into four different genogroups of *Vibrio* sp., with an overlap in one group between the wild and the cultured species. This may suggest a spontaneous transmission between the wild and the farm fish. The first group showed high similarity to *V. parahaemolyticus* and *V. alginolyticus*. According to Montieri et al. [35], these two *Vibrio* species share nearly identical sequences in 16S rRNA gene (99.8% identity), and therefore this gene is inadequate for the separation between the two species. The second group showed high similarity to *V. harveyi*. These species are known to be dangerous to humans, marine fish, and invertebrates and they can cause various diseases including vasculitis, gastroenteritis, septicemia, and skin infections [51,52,53]. The third group contained only one strain that belonged to the Lessepsian fish *S. lessepsianus*, which showed similarity to uncultured *Vibrionaceae* bacterium isolated from pinfish (*Lagodon rhomboids*) [54], with a 95% homology. To the best of our knowledge, there were no records in the literature about the presence of *Vibrio* sp. in this fish species. A possible explanation is that there is species-specific adaptation of some *Vibrio* species [55] and, therefore, this specific genotype might be a new *Vibrio* genotype that is specific for *S. lessepsianus* and possibly other marine fish. Further isolation and characterization of *Vibrio* species is needed from this fish to understand its full genetic properties. In addition, the fourth group contained three identical strains all belonging to the cultured *S. aurata* from 2017 without any similar references. It seems that this group has a unique insertion of 11 nucleotides which is common in 16S rRNA gene sequences, where insertion–deletion events are frequent and result in length differences among homologous sequences [56]. The phylogenetic analysis of *Mycobacterium* sp. reveals a clear separation between the wild and the cultured species: the wild species were positive across two main groups of *Mycobacterium* sp. (with high similarity to *M. peregrinum* and *M. neoaurum*), while positive samples of the cultured fish *S. aurata* were similar to *M. marinum*. All these *Mycobacterium* sp. are known as pathogenic species. Both *M. peregrinum* and *M. neoaurum* are fast growing mycobacteria that can cause bloodstream infections in immunocompromised hosts, and, unlike other NTM species [57]. *M. marinum* is a recognized fish pathogen that can also infect endothermic organisms, including humans. In human infections, *M. marinum* gains access to the body through skin abrasions and generally produces superficial and self-limiting lesions which involve the cooler parts of the body such as hands, forearms, elbows, and knees [58,59]. Although *M. marinum* is a well-recognized pathogen of fish, *M. peregrinum* has only recently been associated with diseases in fish [60]. Even though mycobacteriosis cases have been reported previously in wild and cultured fish species [39,61], in this study, we documented Mycobacteria in *N. randalli* for the first time. Over the last few decades, the 16S rRNA gene has emerged as a good standard for determining phylogenetic relationships of bacteria [62]. By using PCR amplification and direct sequencing of 16S rRNA products, Knibb et al. [63] identified *M. marinum* directly from infected fish. This has allowed both proper taxonomic assignment and has opened the way to molecular epidemiologic analysis at the same time. However, even though this gene is still considered a key standard for bacterial identification [64,65], as more sequence information has accumulated over time, it has become evident that the resolution power of 16S rRNA sequences alone is often insufficient when closely related organisms are compared [66]. Furthermore, Palys et al. [66] suggested that protein-encoding genes may be more discriminative than those encoding rRNA, while the analysis of two or more unlinked loci would prevent bacterial misclassification due to possible homologous recombination with other taxa. Therefore, further molecular analyses are needed to understand the epidemiology and pathogenicity of the *Vibrio* sp. and *Mycobacterium* sp. identified in this study, both on fish and humans.

In the summer of 2018, there was an outbreak of *M. marinum* in three out of the seven fish cages examined. The death rate ranged 5.7–13.5%, and some of the fish that survived showed clinical signs for the pathogen or no signs at all. *M. marinum* was isolated from the kidney and spleen of both juvenile and adult fish. There was no evidence of the disease in 2017. A possible explanation is that in 2017 each cage was populated with ~350,000 fish, in contrast to 2018 where each cage was populated with ~470,000 fish. These conditions might have led to the disease outbreaks. As farmed fish are monitored regularly, they can be used as sentinels to evaluate pathogen exposure in the aquatic environment. However, this approach has utility only if farmed fish are susceptible to the pathogen and enter the marine environment free of the pathogen of interest. In addition, occurrence of disease in farmed fish populations does not necessarily imply occurrence of the same disease in wild populations. It is difficult to evaluate the health effect of escapees on the ecosystem without taking into consideration the qualitative aspects of wild fish assemblages around farms. Cross-contagion between farmed and wild fish species with shared pathogens may occur [67] either through movements of individual fish or through species-specific migrations [68,69]. Connection among farms and other marine areas of interest through wild fish movements have been demonstrated both in Norwegian [70] and in Mediterranean fish farms [67]. Unlike parasitic pathogens, bacteria seem to exhibit higher potential to spread between wild and farmed fish. This is likely because the ecological barriers that exist for parasite transfer do not represent a great obstacle for bacteria [13]. Firstly, bacteria are almost always present on the skin surface of fish. Secondly, bacterial diseases are usually treated by non-professional staff at the farms, and consequently involve increased risk of developing resistance and more pathogenic strains. Finally, bacteria are often generalists and do not need wild conspecifics to spread from farmed fish [13]. There is a potential risk of pathogen transmission through movements of escaped and wild fish in Mediterranean fish farming areas, but actual transmission has been documented only in a handful of cases [71]. Due to technical or operational malfunctions, infected farmed fish may escape and, in theory, spread pathogens to other cages/farms and wild fish as well. Furthermore, infected wild fish might also transfer pathogens to the farmed fish [72]. This co-infection process leads to a large variety of shared pathogens among wild and farmed fish, while the various pathways of pathogen transmission increase the potential for infection and render epidemiological risk management difficult [71]. Further research on molecular mechanisms of disease transmission in aquaculture and marine environments, as well more holistic analyses of pathogenic events in the Mediterranean Sea, are needed to clarify the potential of transmission of pathogens from aquaculture to the marine ecosystems.

## 5. Conclusions

As Mediterranean mariculture is expected to increase in the near future, bacteria such as *Vibrio* sp. and *Mycobacterium* sp. remain important infectious pathogens of wild and cultured finfish and should be extensively studied and investigated. *Vibrio* sp. and *Mycobacterium* sp. prevalence in asymptomatic fish may indicate that they can serve as carriers and infect other susceptible species. As many *Vibrio* sp. and *Mycobacterium* sp. infect fish and humans, the potential for zoonotic infection presents an additional challenge. Although these diseases have been studied in fish for over a century, basic questions about their pathobiology, including transmission and host defense mechanisms remain unknown. Additionally, effective prophylaxis, control measures, and non-lethal diagnostics require research and development. However, with the advent of modern molecular detection methods, epidemiological techniques, and vaccinology, considerable potential exists for the improvement of our understanding and control of those diseases in the future.

## Figures and Tables

**Table 1:** Wild fish organisms sampled and analyzed in this study. Total number of specimens tested and number of wild fish sampled from each sampling site in 2016 and 2017.

Year	Family, Species	Origin *	Acre	Kishon	Jaffa	Ashdod	Total
2016	*Mulidae*						
	*Mullus surmuletus*	m	3	13	3	3	22
	*Nemipteridae*						
	*Nemipterus randalli*	l	3	13	10	3	29
	*Synodonitidae*						
	*Saurida lessepsianus*	l	9	13	13	3	38
	*Clupeidae*						
	*Sardinella aurita*	m	3	11	10	nc	24
2017	*Mulidae*						
	*Mullus surmuletus*	m	nc	15	nc	nc	15
	*Nemipteridae*						
	*Nemipterus randalli*	l	nc	15	15	12	42
	*Synodonitidae*						
	*Saurida lessepsianus*	l	nc	nc	15	nc	15
	*Clupeidae*						
	*Sardinella aurita*	m	nc	nc	20	5	25

* m, Mediterranean natives; l, Lessepsian migrants; nc, not collected.

**Table 2:** Cultured *Sparus aurata* sampled and analyzed in this study. Total number of specimens tested and number of cultured fish farm sampled in 2017 and 2018.

Family, Species	Origin *	2017	2018	Total
*Sparidae*				
*Sparus aurata*	m, f	45	27	72

* m, Mediterranean natives; f, farmed species.

**Table 3:** Relative distribution and percentage of *Vibrio* in kidney and liver tissues from wild and cultured fish. Only specimens tested for both tissues were included in the statistical analyses.

Fish Species	Kidney Tissue		Liver Tissue	
	N	%	N	%
*Mullus surmuletus*	2/37	4.5	2/37	4.5
*Nemipterus randalli*	6/62	7.9	0/62	0.0
*Sardinella aurita*	2/44	5.4	2/44	5.4
*Saurida lessepsianus*	1/47	1.2	0/47	0.0
*Sparus aurata*	2/61	3.3	3/61	9.4
Total	13/251	2.5	7/251	8.2

**Table 4:** Relative distribution and percentage of *Vibrio* in juvenile and adult wild and cultured fish.

Fish Species	Positive for Vibrio			
	Mature		Juvenile	
	N	%	N	%
*Nemipterus randalli*	6/48	12.5	0/23	0
*Sardinella aurita*	2/33	6.1	0/16	0
*Saurida lessepsianus*	0/49	0	1/4	25
*Sparus aurata*	1/20	5	4/52	7.7
Total	9/150	6	5/59	5.3

**Table 5:** *Vibrio* sp. prevalence in wild fish species from the Mediterranean Sea. The results are based on the PCR targeting 16S rRNA segments. Positive results refer to one or more isolated tissue.

Fish Species	2016			2017			Total		
	N	Positive	% Positive	N	Positive	% Positive	N	Positive	% Positive
*Mullus surmuletus*	22	2	9.09	15	2	13.33	37	4	10.81
*Sardinella aurita*	24	4	16.67	25	0	0.00	49	4	8.16
*Saurida lessepsianus*	38	1	2.63	15	0	0.00	53	1	1.89
*Nemipterus randalli*	29	6	20.69	42	0	0.00	71	6	8.45
Total wild	113	13	11.50	97	2	2.06	210	15	7.14

**Table 6:** *Vibrio* prevalence in the cultured fish *S. aurata* from the Mediterranean Sea farmed fish. The results are based on PCR targeting 16S rRNA segments. Positive result refers to one or more isolated tissue.

Fish Species	2017			2018			Total		
	N	Positive	% Positive	N	Positive	% Positive	N	Positive	% Positive
*Sparus aurata*	45	4	8.89	27	1	3.70	72	5	6.94

**Table 7:** *Mycobacterium* prevalence in wild fish species from the Mediterranean Sea. The results are based on PCR targeting 16S rRNA segments.

Fish Species	2016			2017			Total		
	N	Positive	% Positive	N	Positive	% Positive	N	Positive	% Positive
*Mullus surmuletus*	22	0	0	15	1	6.67	37	1	2.7
*Sardinella aurita*	24	0	0	25	0	0	49	0	0
*Saurida lessepsianus*	38	0	0	15	0	0	53	0	0
*Nemipterus randalli*	29	0	0	42	4	9.52	71	4	5.63
Total wild	113	0	0	97	5	5.15	210	5	2.38

**Table 8:** *Mycobacterium* prevalence in the cultured fish *S. aurata* from the Mediterranean Sea fish farm. The results are based on PCR targeting 16S rRNA segments.

Fish Species	2017			2018			Total		
	N	Positive	% Positive	N	Positive	% Positive	N	Positive	% Positive
*Sparus aurata*	45	0	0	27	5	18.52	72	5	6.94

**Table 9:** Relative distribution and percentage of *Mycobacterium* sp. in kidney and liver tissues from wild and cultured fish. Only specimens tested for both tissues were included in the statistical analysis.

Fish Species	Positive for *Mycobacterium*			
	Kidney Tissue		Liver Tissue	
	N	%	N	%
*Mullus surmuletus*	1/37	2.7	0/37	0
*Nemipterus randalli*	4/62	6.5	2/62	3.2
*Sardinella aurita*	0/44	0	0/44	0
*Saurida lessepsianus*	0/47	0	0/47	0
*Sparus aurata*	2/61	3.3	0/61	0
Total	7/251	2.8	2/251	0.8

**Table 10:** Relative distribution and percentage of *Mycobacterium* in juvenile and adult wild and cultured fish.

Fish Species	Positive for *Mycobacterium*			
	Mature		Juvenile	
	N	%	N	%
*Nemipterus randalli*	5/48	10.4	0/23	0
*Sardinella aurita*	0/33	0	0/16	0
*Saurida lessepsianus*	0/49	0	0/4	0
*Sparus aurata*	2/20	10	3/52	5.8
Total	7/150	4.7	3/95	3.2

## Supplementary Materials

The following are available online at https://www.mdpi.com/2076-2607/8/6/863/s1. Table S1: List of *Mycobacterium* sp. positive results with GenBank accession numbers, Table S2: List of *Vibrio* sp. positive results with GenBank accession numbers.
