# Correction: Regev, Y., et al. Molecular Identification and Characterization of *Vibrio* Species and *Mycobacterium* Species in Wild and Cultured Marine Fish from the Eastern Mediterranean Sea. *Microorganisms* 2020, *8*, 863

**DOI:** 10.3390/microorganisms8081153

**Published:** 2020-07-30

**Authors:** Yael Regev, Nadav Davidovich, Ran Berzak, Stanley C. K. Lau, Aviad P. Scheinin, Dan Tchernov, Danny Morick

**Affiliations:** 1 Department of Marine Biology, Leon H. Charney School of Marine Sciences, University of Haifa, Haifa 3498838, Israel; yaelchac@gmail.com (Y.R.); ranberzak@gmail.com (R.B.); shani.aviad@gmail.com (A.P.S.); dtchernov@univ.haifa.ac.il (D.T.); 2 Morris Kahn Marine Research Station, University of Haifa, Haifa 3498838, Israel; 3 Israeli Veterinary Services, Bet Dagan 5025001, Israel; Nadavd@moag.gov.il; 4 Department of Ocean Science, The Hong Kong University of Science and Technology, Clear Water Bay, Kowloon, Hong Kong; scklau@ust.hk

## Change in Affiliations

We would like to change the authors’ affiliation of paper [1] from:

Yael Regev ^1,2^, Nadav Davidovich ^3^, Ran Berzak ^1,2^, Stanley C. K. Lau ^4^, Aviad P. Scheinin ^1,2^, Dan Tchernov ^1,2^ and Danny Morick ^1,2,^*

^1^  Department of Marine Biology, Leon H. Charney School of Marine Sciences, University of Haifa, Haifa 3498838, Israel; yaelchac@gmail.com (Y.R.); ranberzak@gmail.com (R.B.); shani.aviad@gmail.com (A.P.S.); dtchernov@univ.haifa.ac.il (D.T.)
^2^  Morris Kahn Marine Research Station, University of Haifa, Haifa 3498838, Israel
^3^  Israeli Veterinary Services, Bet Dagan 5025001, Israel; Nadavd@moag.gov.il
^4^  Department of Ocean Science, The Hong Kong University of Science and Technology, Clear Water Bay, Kowloon, Hong Kong; scklau@ust.hk
*  Correspondence: dmorick@univ.haifa.ac.il

to the correct version, as follows:

Yael Regev ^1,2^, Nadav Davidovich ^3^, Ran Berzak ^1,2^, Stanley C. K. Lau ^4,5^, Aviad P. Scheinin ^1,2^, Dan Tchernov ^1,2,5^ and Danny Morick ^1,2,5,^*

^1^  Department of Marine Biology, Leon H. Charney School of Marine Sciences, University of Haifa, Haifa 3498838, Israel; yaelchac@gmail.com (Y.R.); ranberzak@gmail.com (R.B.); shani.aviad@gmail.com (A.P.S.); dtchernov@univ.haifa.ac.il (D.T.)
^2^  Morris Kahn Marine Research Station, University of Haifa, Haifa 3498838, Israel
^3^  Israeli Veterinary Services, Bet Dagan 5025001, Israel; Nadavd@moag.gov.il
^4^  Department of Ocean Science, The Hong Kong University of Science and Technology, Clear Water Bay, Kowloon, Hong Kong; scklau@ust.hk
^5^  Hong Kong Branch of Southern Marine Science and Engineering Guangdong Laboratory (Guangzhou), Hong Kong, China
*  Correspondence: dmorick@univ.haifa.ac.il

We apologize for any inconvenience caused to the readers.
