# Supplementary material for: Molecular Identification and Characterization of Vibrio Species and Mycobacterium Species in Wild and Cultured Marine Fish from the Eastern Mediterranean Sea
Source: Microorganisms. 2020 Jun 7;8(6):863. doi: 10.3390/microorganisms8060863 (PMC7356242; doi:10.3390/microorganisms8060863)
Supplement: Supplementary file 1 [file microorganisms-08-00863-s001.pdf]

Table S1. List of *Mycobacterium* sp. positive results with GenBank accession numbers.

Host species	Tissue	Year	Accession number
Nemipterus randalli	Liver	2017	MN602974
Nemipterus randalli	Kidney	2017	MN602975
Nemipterus randalli	Liver	2017	MN602976
Mullus surmuletus	Kidney	2017	MN602977
Nemipterus randalli	Kidney	2017	MN602978
Nemipterus randalli	Kidney	2017	MN602979
Sparus aurata	Kidney	2018	MN602980
Sparus aurata	Kidney	2018	MN602981
Sparus aurata	Spleen	2018	MN602982
Sparus aurata	Spleen	2018	MN602983
Sparus aurata	Spleen	2018	MN602984

Table S2. List of *Vibrio* sp. positive results with GenBank accession numbers.

Host species	Tissue	Year	Accession number
Saurida lessepsianus	Kidney	2016	MN602985
Mullus surmuletus	Kidney	2016	MN602986
Mullus surmuletus	Kidney	2016	MN602987
Nemipterus randalli	Kidney	2016	MN602988
Nemipterus randalli	Kidney	2016	MN602989
Nemipterus randalli	Kidney	2016	MN602990
Nemipterus randalli	Kidney	2016	MN602991
Nemipterus randalli	Kidney	2016	MN602992
Nemipterus randalli	Kidney	2016	MN602993
Sardinella aurita	Kidney	2016	MN602994
Sardinella aurita	Liver	2016	MN602995
Sardinella aurita	Kidney	2016	MN602996
Sardinella aurita	Liver	2016	MN602997
Sparus aurata	Liver	2017	MN602998
Mullus surmuletus	Liver	2017	MN602999
Mullus surmuletus	Liver	2017	MN603000
Sparus aurata	Liver	2017	MN603001
Sparus aurata	Kidney	2017	MN603002
Sparus aurata	Liver	2017	MN603003
Sparus aurata	Kidney	2018	MN603004

© 2020 by the author. Licensee MDPI, Basel, Switzerland. This article is an open access article distributed under the terms and conditions of the Creative Commons Attribution (CC BY) license (http://creativecommons.org/licenses/by/4.0/).
